# Diagnosis of Methionine/Valine Variant Creutzfeldt-Jakob Disease by Protein Misfolding Cyclic Amplification

**DOI:** 10.3201/eid2407.172105

**Published:** 2018-07

**Authors:** Daisy Bougard, Maxime Bélondrade, Charly Mayran, Lilian Bruyère-Ostells, Sylvain Lehmann, Chantal Fournier-Wirth, Richard S. Knight, Robert G. Will, Alison J.E. Green

**Affiliations:** Etablissement Français du Sang, Montpellier, France (D. Bougard, M. Bélondrade, C. Mayran, L. Bruyère-Ostells, C. Fournier-Wirth);; University of Montpellier, Montpellier (S. Lehmann);; University of Edinburgh, Edinburgh, Scotland, UK (R.S. Knight, R.G. Will, A.J.E. Green)

**Keywords:** Creutzfeldt-Jakob disease, CJD, variant Creutzfeldt-Jakob disease, vCJD, sporadic Creutzfeldt-Jakob disease, sCJD, genetic Creutzfeldt-Jakob disease, gCJD, methionine/valine variant, cerebrospinal fluid, CSF, prions and related diseases, protein misfolding cyclic amplification, PMCA, diagnosis

## Abstract

A patient with a heterozygous variant of Creutzfeldt-Jakob disease (CJD) with a methionine/valine genotype at codon 129 of the prion protein gene was recently reported. Using an ultrasensitive and specific protein misfolding cyclic amplification–based assay for detecting variant CJD prions in cerebrospinal fluid, we discriminated this heterozygous case of variant CJD from cases of sporadic CJD.

Variant Creutzfeldt-Jakob disease (vCJD) is a neurodegenerative infectious disease caused by transmission of a cattle prion disease (bovine spongiform encephalopathy) to humans (*1*). Most vCJD cases have occurred in the United Kingdom, where an estimated 1 in 2,000 persons is potentially asymptomatically infected, although there is some uncertainty about interpretation of detection of abnormal prion protein (PrP^TSE^) in appendix tissues on which this incidence is based (*2*) (Public Health England, https://assets.publishing.service.gov.uk/government/uploads/system/uploads/attachment_data/file/546883/hpr2616.pdf).

Until recently, all clinical cases of vCJD for which the prion protein gene has been analyzed have been shown to be methionine homozygous at codon 129, a genotype present in almost 40% of Caucasian populations. The report of the first definite heterozygous methionine/valine vCJD patient who died in 2016 (*3*) underlined previous concern about a possible second wave of vCJD cases (*4*). The clinical features of this patient were more similar to those of patients with sporadic CJD (sCJD) than to those with vCJD. This patient had met the agreed surveillance diagnostic criteria for probable sCJD (*5*). However, vCJD was diagnosed during an autopsy; florid plaques were observed by histologic examination of the brain and peripheral detection of PrP^TSE^ in lymphoid tissues. Western blot analysis of brain tissue confirmed a type 2B molecular profile of PrP^TSE^, which is characteristic for vCJD.

A diagnostic test to identify methionine/valine heterozygous vCJD cases is urgently needed to enable discrimination between heterozygous vCJD and sCJD and in view of the potential reservoir of methionine/valine heterozygous asymptomatic vCJD carriers in the blood donor population. We developed a highly sensitive and specific assay that accurately detects vCJD prions in blood even before the occurrence of clinical signs (*6*). We adapted this assay, which was based on protein misfolding cyclic amplification (PMCA) (*7*), for specific detection of vCJD in cerebrospinal fluid (CSF) and confirmed the ability of this assay to differentiate patients with atypical heterozygous vCJD from patients with sCJD.

## The Study

We blindly analyzed 98 CSF samples provided by the National CJD Research and Surveillance Unit (Edinburgh, Scotland, UK) and the Centre Hospitalier Universitaire de Montpellier (Montpellier, France) after obtaining appropriate consent. Clinicians distributed CSF samples into blinded panels from the United Kingdom and France; 41 from patients with vCJD; 23 from patients with sCJD; 1 from a patient with genetic CJD; and 33 from patients with non-CJD, including samples from patients with Alzheimer’s disease and patients with nonneurodegenerative diseases.

CSF samples were thawed at room temperature and used directly in PMCA. We performed PMCA amplification by using brains from humanized transgenic mice as substrate for normal prion protein. After successive rounds of 160 cycles of PMCA for 15 min and sonication for 20 s, we detected PrP^TSE^ by using Western blot after digestion with proteinase K (*6*).

Of the 98 CSF samples analyzed, our assay identified 40 of 41 cases of clinical vCJD, including the methionine/valine heterozygous patient, thus showing a diagnostic sensitivity of 97.6% (95% CI 87.1%–99.9%) (Table). One CSF sample from a probable case of vCJD showed a negative result. After decoding by clinicians, we retested this sample in duplicate; it showed a positive result.

**Table:** Analysis of CSF samples from patients with CJD and controls by PMCA*

Diagnosis	No. patients with positive detection of PrP^TSE^ in CSF and codon 129 genotype/no. tested				Analytical performance, % (95% CI)
	Total	MM	MV	VV	
Clinical CJD					
Variant CJD	40/41†	37/38	1/1	NA	Diagnostic sensitivity 97.6 (87.1–99.9)
Definite	29/29	28/28	1/1	NA	
Probable	10/11	8/9	NA	NA	
Possible	1/1	1/1	NA	NA	
Sporadic CJD	0/23†	0/7	0/12	0/3	Analytic specificity 100 (93.7–100)
Definite	0/14	0/2‡	0/10	0/1‡	
Probable	0/9	0/5	0/2	0/2	
Genetic CJD	0/1	0/1	NA	NA	Analytic specificity 100 (93.7–100)
Non-CJD					Analytic specificity 100 (93.7–100)
Alzheimer’s disease	0/12	ND	ND	ND	
Other nonneurodegenerative diseases	0/21	ND	ND	ND	

*CJD, Creutzfeldt-Jakob disease; CSF, cerebrospinal fluid; MM, methionine homozygous; MV, methionine/valine heterozygous; NA, not available; ND, not determined; PMCA, protein misfolding cyclic amplification; PrP^TSE^, abnormal prion protein; VV, valine homozygous. †Genotyping of the prion protein gene at codon 129 was not conducted for 2 patients with variant CJD and 1 patient with sporadic CJD. ‡The protease-resistant protein subtype was available for 3 patients with definite sporadic CJD and showed an equal distribution of MM1, MM2a, and VV2a.

Our assay also showed high analytical specificity; 0 of 57 potentially cross-reacting CSF specimens from patients with sCJD, gCJD, Alzheimer's disease, and other nonneurodegenerative diseases showed a positive result (specificity 100% [95% CI 93.7%–100%]) (Table). The case-patient with methionine/valine heterozygous vCJD was specifically discriminated from the 12 methionine/valine heterozygous neuropathologically confirmed sCJD case-patients tested.

We then compared by using Western blot the PrP^TSE^ molecular signature obtained for the clinical vCJD amplified samples from classical methionine homozygous cases and the new methionine/valine heterozygous vCJD case with that of the reference brain sample from a patient with vCJD (Figure). As expected, the profile obtained after PMCA amplification of the CSF from the methionine/valine heterozygous vCJD patient was similar to those obtained for methionine homozygous vCJD patients. The characteristic type 2 mobility and clear predominance of the diglycosylated isoform was obtained for all vCJD patients before or after amplification.

**Figure:**
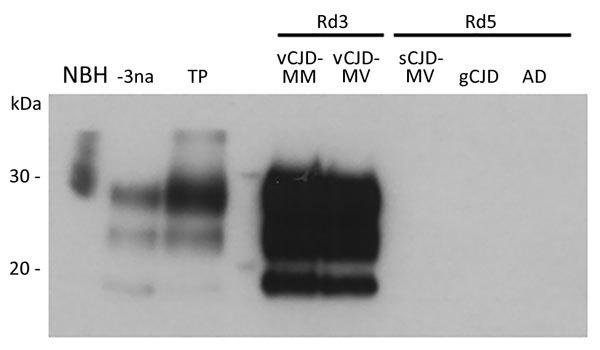
Western blot analysis of vCJD prions obtained after amplification by protein misfolding cyclic amplification (PMCA) of cerebrospinal fluid (CSF) from 2 patients with vCJD (MM and MV) and 3 control patients and a crude reference brain homogenate from a vCJD patient (National Institute for Biological Standards and Control [Ridge, UK] no. NHBY0/0003). Abnormal prion protein patterns were assessed by using antibody 3F4 after digestion of samples with proteinase K. A total of 20 μL of each sample was subjected to electrophoresis on a 12% polyacrylamide gel. Lane NBH, negative control brain homogenate from a person without CJD and no digestion with proteinase K (National Institute for Biological Standards and Control no. NHBZ0/0005); lane -3na, Western blot control (10^−3^ dilution of vCJD reference brain sample without amplification); lane TP, positive control for amplification (10^−6^ dilution of vCJD reference brain sample after 1 round of PMCA); lane vCJD-MM, CSF from a patient with MM vCJD after 3 rounds of PMCA; lane vCJD-MV, CSF from a patient with MV vCJD after 3 rounds of PMCA; lane sCJD-MV, CSF from a patient with MV sCJD after 5 rounds of PMCA; lane gCJD, CSF from a patient with gCJD after 5 rounds of PMCA; lane AD, CSF from a patient with Alzheimer’s disease after 5 rounds of PMCA. CJD, Creutzfeldt-Jakob disease; gCJD, genetic CJD; MM, methionine homozygous; MV, methionine/valine heterozygous; Rd, round (1 round indicates 80 cycles of PMCA); sCJD, sporadic CJD; vCJD, variant CJD.

## Conclusions

We report a specific detection method that enables clinical diagnosis of a heterozygous methionine/valine heterozygous vCJD patient. This patient was the first definite heterozygous patient described since the start of the vCJD epidemic in the United Kingdom in 1996 (*3*). Clinical diagnosis was difficult because clinical signs and symptoms, particularly cerebral appearance by magnetic resonance imaging, were suggestive of sCJD (*3*). The vCJD blood test (direct detection assay) developed by the Medical Research Council Prion Unit (London, UK) (*8*) showed a negative result for this case-patient. We found characteristic vCJD prion protein amplification in the CSF, which led to a specific diagnosis of vCJD because sCJD samples did not show positive results by PMCA. This result also demonstrates the possibility of amplifying methionine/valine heterozygous vCJD prion protein by PMCA with a substrate from humanized transgenic mice that overexpress homozygous methionine prion protein (*9*). However, PMCA analysis should be performed in a Biosafety Level 3 laboratory and requires highly experienced personnel.

Iatrogenic transmission of vCJD by blood transfusion has been documented in 3 recipients of nonleukodepleted erythrocyte concentrates from blood donors during development of disease (*10*). One additional probable case of vCJD transmission by blood transfusion was identified during an autopsy of a methionine/valine heterozygous patient who died from a nonneurologic disorder and in whom vCJD prion protein was detected in the spleen (*11*). The presence of infectivity in blood of the definite methionine/valine heterozygous vCJD patient involved in our study is uncertain and requires further investigation.

From a clinical point of view, prion amplification technologies, such as PMCA and real-time quaking-induced conversion (RT-QuIC), have already shown their sensitive detection of disease-related prion protein in biologic fluids (*6**,**12**–**14*). Independent studies have shown that detection of prion protein seeding activity in CSF by RT-QuIC might have a specificity of 99%–100% for diagnosis of sCJD (*13**,**15*). Application of RT-QuIC and PMCA for CSF samples might represent a suitable strategy for premortem discrimination between sCJD and vCJD including methionine/valine heterozygous case-patients, particularly for cases with a heterozygous codon 129 genotype in which clinical distinction between sCJD and vCJD is problematic.
